# Microsatellite diversity in four cultivated species of *Actinidiaceae* and *Rutaceae*

**DOI:** 10.6026/97320630019230

**Published:** 2023-03-31

**Authors:** Simerpreet Kaur, Prakash Chand Sharma

**Affiliations:** 1 University School of Biotechnology, Guru Gobind Singh Indraprastha University, New Delhi, India

**Keywords:** Microsatellites, Relative abundance, Relative density, Comparative genomics, GC content

## Abstract

Microsatellites or Simple Sequence Repeats (SSRs) are short iterations of 1-6 bp in the genomes of almost all living organisms. Our study aimed to explore the microsatellite diversity in four cultivated species, namely *Actinidia* chinensis, *Actinidia eriantha*, *Citrus maxima*, and *Citrus sinensis* of the *Actinidiaceae* and *Rutaceae* families. We present a comprehensive analysis of microsatellite abundance, distribution, and motif composition in the genomes of these species. The association of microsatellite abundance with genomic features such as genome size, GC content, number of microsatellites, relative abundance, and relative density was also examined. The results revealed significant variations in the frequency and distribution of microsatellites across the genomes of these four species. Notably, a positive correlation was observed between genome size and microsatellite number as well as with GC content, indicating that larger genomes provide more opportunities for the accumulation of microsatellites. Furthermore, a negative correlation of genome size with relative microsatellite abundance and relative density was observed. These findings provide new insights into the microsatellite landscape of *Actinidiaceae* and *Rutaceae*, which could be explored for the development of microsatellite markers for diverse applications in the characterization of genetic diversity, molecular plant breeding, and phylogenetic analysis.

## Background:

Microsatellites, also known as simple sequence repeats (SSRs), are short tandem repeats of DNA motifs that are widely distributed in the genomes of almost all organisms [1]. Due to their high degree of polymorphism and co-dominant inheritance, microsatellites have become a popular marker system for diverse applications, including genetic diversity analysis, population genetics, and molecular plant breeding [2]. However, despite their widespread use, many plant species have not been extensively studied for microsatellite-related features present in their genomes. In this study, we have included four plant species from two important plant families, namely *Actinidiaceae* and *Rutaceae*. The *Actinidiaceae* family includes Actinidia chinensis and *Actinidia eriantha*, two closely related kiwifruit species that are native to China and have potential in commercial fruit production [3]. The species from the *Rutaceae* family included *Citrus maxima* and *Citrus sinensis*, two citrus fruit species that are widely grown around the world [4]. The *Actinidiaceae* and *Rutaceae* families are of significant ecological and economic importance, containing a diverse range of species. The *Actinidiaceae* family, also known as the kiwifruit family, comprises approximately 70 species of woody vines and shrubs that are cultivated for their edible fruit [5]. *Actinidia chinensis* and *Actinidia eriantha* are two important cultivated species within this family, known for their high nutritional value and potential health benefits [6, 7]. On the other hand, *Rutaceae*, commonly known as the citrus family, comprises around 1600 species of trees, shrubs, and herbs, many of which are cultivated for their fruit, medicinal properties, or ornamental value [8]. *Citrus maxima* and *C. sinensis* are two economically important cultivated species within this family, known for their juicy and flavoured fruit that is consumed worldwide [9]. Both *Actinidiaceae* and *Rutaceae* have been subject to extensive genetic research due to their ecological and economic importance, however, no detailed study is available on microsatellite dynamics [10]. We aimed to identify and characterize the distribution of microsatellites in these four species using a bioinformatics approach. Our analysis focused on the identification of microsatellite motifs, the number and distribution of microsatellite loci, and their motif type. The results of this study will provide valuable information on the comparative analysis of the distribution of microsatellites and their association with some genomic features like chromosome number, genome size, and GC content. The critical comparative analysis of microsatellite variation within these families offers valuable insights into the evolutionary processes shaping genetic diversity. This analysis also has potential applications for developing molecular markers to improve crops and conserve genetic diversity [11]. Furthermore, this study will contribute to the growing body of knowledge on microsatellite dynamics in plant genomes and its role in genome evolution.

## Material and Methods:

## Genome acquisition:

The whole genome sequences of the four species included in the study were downloaded from the latest sequence assemblies from the NCBI FTP server [12]. The NCBI taxonomy browser was used to obtain data about each species, as outlined in Table 1 [13]. The downloaded sequences were in FASTA format and were used for the subsequent analysis of microsatellite distribution at the genomic level.

## Detection, screening, and study of microsatellites:

A PERL script MIcroSAtellite was used for the identification of microsatellites in the whole genome sequences and coding region of plant genomes [14]. The software was used to identify the presence of microsatellites, and perfect microsatellites were selected using the following criteria: mononucleotide with ≥ 10 repeats, dinucleotide with ≥ 6 repeats, trinucleotide with ≥ 5 repeats, tetranucleotide with ≥ 5 repeats, pentanucleotide with ≥ 5 repeats, and hexanucleotide with ≥ 5 repeats. The default settings were kept for the rest of the parameters. For analysis purposes, reverse compliments of microsatellite motifs and unit patterns of circular permutations were considered as one type [15, 16].

## Quantification of relative abundance and relative density of microsatellites:

To facilitate interspecies comparison, the total microsatellites were normalized as relative abundance (RA) and relative density (RD). The RA was determined as the number of microsatellites per Mb of the genome, while the RD was determined as the total length of microsatellites per Mb of the genome.

## Correlation between Genome Size, GC Content, and Microsatellite Features:

To assess the relationship between relative abundance, relative density, genome size, and GC content, we conducted statistical significance analysis using the cor.test () function with the method = "pearson" in the R programming environment. Plots were generated using MS-excel and ggplot2, an elegant graphics for data analysis package within the R programming language and environment (version 4.1.0) facilitated by RStudio, an Integrated Development Environment) (R core team, 2021) [17, 18,19].

## Results and Discussion:

**Figure 1:**
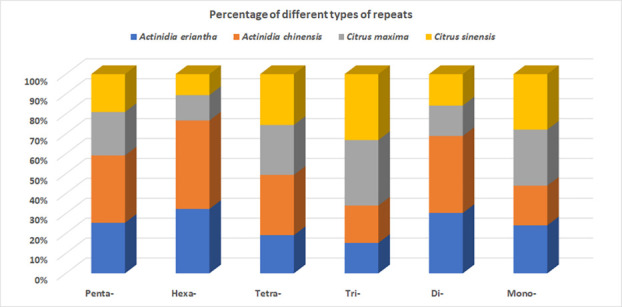
Distribution of percentage of different types of repeats in four cultivated species: *Actinidia chinensis*, Actinidia eriantha, *Citrus maxima*, and *Citrus sinensis*. The types of repeats include mono-, di-, tri-, tetra-, penta-, and hexa-nucleotide repeats

We investigated the microsatellite dynamics of four different plant species belonging to the *Actinidiaceae* and Rutacecae families. A total of 1011108 microsatellites were mined from the whole genome sequences of the four species, and their relative abundance and density were analysed to allow interspecies comparisons. The analysis included an examination of GC content, as well as the determination of the percentage of various repeat types. The results showed that the number of microsatellites varied among the four species, with A. eriantha having the highest number of microsatellites (381748), followed by *A. chinensis* (296474), *C. maxima* (189367), and *C. sinensis* (143519). The relative abundance and relative density of microsatellites were found to be higher in *C. maxima* and *C. sinensis* compared to *A. eriantha* and *A. chinensis* (Table 1). The higher number of microsatellites in *A. chinensis* and *A. eriantha* may be attributed to their larger genome size, as reported earlier [20]. The percentage of different types of repeats was also determined, and the most abundant repeat type was found to be mononucleotide, followed by, dinucleotide, trinucleotide, tetranucleotide, pentanucleotide, and hexanucleotide. The abundance of different types of repeats varied among the four species, with *A. chinensis* and *A. eriantha* having the highest percentage of dinucleotide repeats, 40.41 and 31.7, respectively whereas *C. maxima* and *C. sinensis* had the highest percentage of trinucleotide repeats, 9.99 and 10.7, respectively) (Figure 1). The present study also investigated the different types of motifs present in the genomic region. The results represented in Table 2, showed that the most common motifs were A/T, AG/CT, AT/AT, and AC/GT, as observed in other plant species also. The AG/CT motif was found to be the most common motif in *A. chinensis*, and *A. eriantha*, whereas the A/T motif was the most common in *C. maxima* and *C. sinensis*. The differences in the frequency of different motifs among the four species may be due to their different evolutionary histories and ecological niches [21].

**Figure 2:**
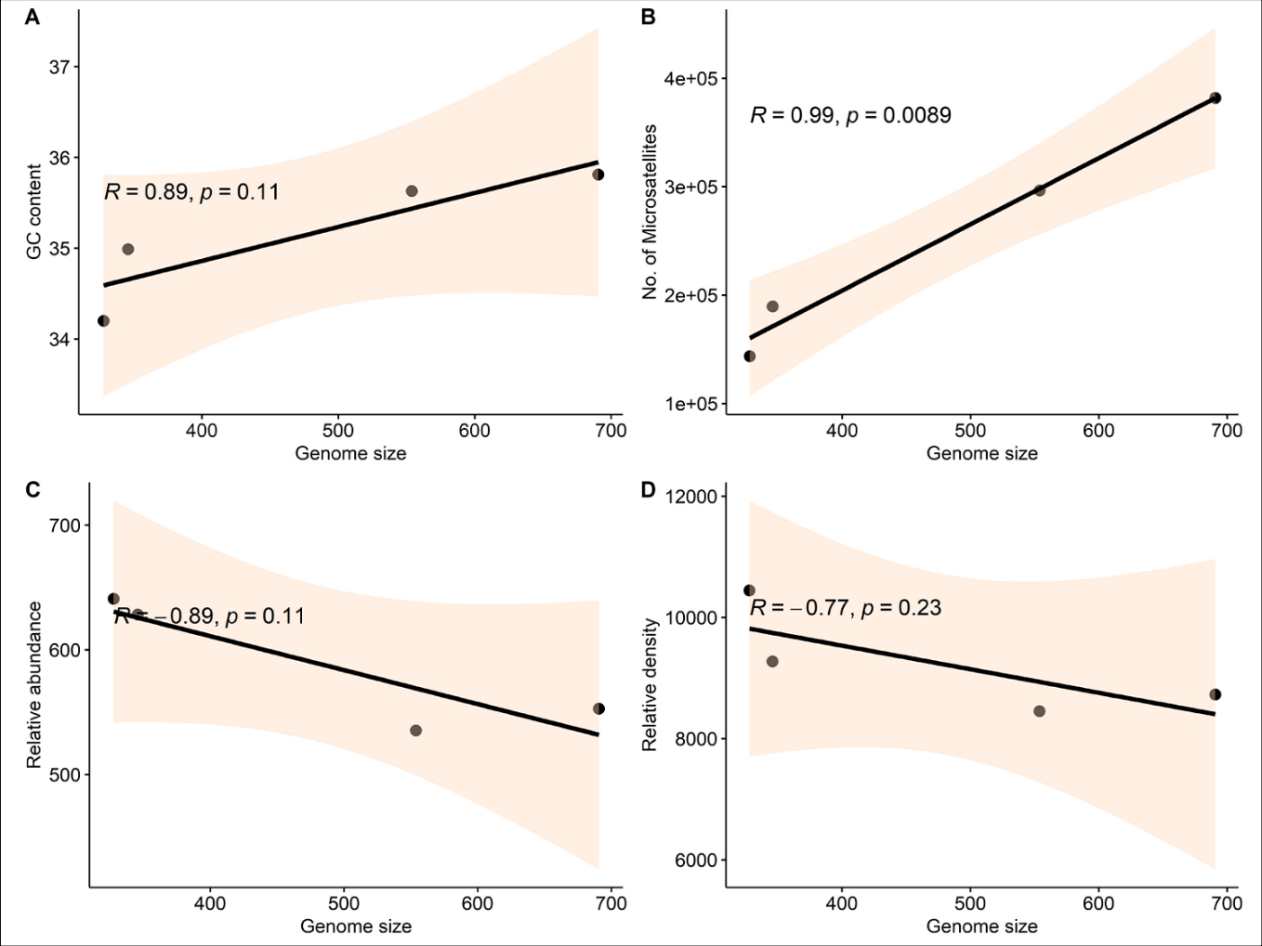
Correlation plot showing the relationship between genome size with GC content, number of microsatellites, relative abundance, and relative density in four species of Actinidia and *Citrus*. Each point represents a species. The correlation coefficients and p-values are shown in the upper left corner of each plot. The black line represents the linear regression line, and the shaded region represents the 95% confidence interval for the regression line. The x-axis and y-axis labels indicate the variables being plotted.

Further, in our study of four species of genera Actinidia and Citrus, we found a positive correlation between genome size and GC content with the number of microsatellites. This positive correlation suggests that species with larger genomes tend to have higher GC content and more microsatellites. One possible explanation for this could be that larger genomes have more space to accommodate these repetitive DNA sequences, which are known to be rich in GC content [22]. Secondly, in larger genomes, longer recombination/replication process allows increased variation in microsatellite regions due to hypervariable nature of these microsatellite regions leading to the rearrangement of microsatellite tracts [23]. Additionally, organisms characterized by a greater GC content tend to possess a reduced number of microsatellites in proportion to their genome magnitude. This can be elucidated by nthe notion that regions abundant in GC exhibit more stability and exhibit lesser susceptibility to errors during DNA replication, thus mitigating the requirement for repair mechanisms [24]. A negative correlation existed between the relative abundance and relative density of microsatellites with genome size (Figure 2). Notably, the correlations between genome size, GC content, and microsatellite abundance exhibited some variation among the four species examined. Specifically, *C. sinensis* displayed the smallest genome size and GC content, yet exhibited the highest relative abundance and density of microsatellites. On the other hand, *A. chinensis* possessed a larger genome size and highest GC content, yet demonstrated the lowest relative abundance and density of microsatellites. *A. eriantha*, however, had the highest genome and *C. maxima* fell in between those two extremes. These variations could be the result of various events during the evolutionary history and genetic makeup of these species. For example, *A. chinensis* is known to have undergone a recent whole-genome duplication event, which could have contributed to its lower genome size than *A. eriantha*. *C. sinensis*, on the other hand, has a long history of domestication and selection, which could have led to the accumulation of more abundance of repetitive DNA sequences and higher GC content [25].

## Conclusion:

The current investigation yields significant perspectives into the microsatellite patterns of four crucial plant families. The outcomes propose that the frequency and distribution of microsatellites are exclusive to each species and exhibit considerable variation among diverse species. The study also sheds light on the distribution of different types of motifs and repeats across the chromosomes, which can be useful for future genetic mapping and marker development studies. The analysis of different types of motifs presents in the genomic region showed that AG/CT was the most frequent motif type among dinucleotide repeats, while AAT/ATA/ATT was the most frequent motif type among trinucleotide repeats. In tetranucleotide repeats, AAAT/ATAA/TAAA was the most frequent motif type. Moreover, the negative correlation between the genome size and the relative abundance and relative density of microsatellites highlights the impact of genomic characteristics on microsatellite dynamics. These findings can have important implications for understanding the mechanisms of genome evolution and genetic diversity in these economically important plant families.

## Figures and Tables

**Table 1:** Detailed information on the genomes of four plant species used in the study

**Name**	**Assembly**	**Family**	**Haploid Chromosome number**	**Genome size (Mb)**	**No. of Microsatellites**	**Relative abundance**	**Relative De**
*Actinidia chinensis*	Red5_PS1_1.69	*Actinidiaceae*	29	553.842	296474	535.3	8452.87
*Actinidia eriantha*	ASM415031v1	*Actinidiaceae*	29	690.61	381748	552.77	8726.58
*Citrus maxima*	ASM200692v1	*Rutaceae*	9	345.757	189367	628.27	9274.5
*Citrus sinensis*	Citrus_sinensis_v1.0	*Rutaceae*	9	327.83	143519	640.98	10443.45

**Table 2:** Abundance of microsatellite motifs in four plant species of *Actinidiaceae* and Rutacecae

**TypesofMotifs**	***A.chinensis***	***A.eriantha***	***C.maxima***	***C.sinensis***
A	281.08	341.86	434.72	447.97
C	4.11	30.7	17.79	10.29
AC	26.1	22.64	20.72	23.27
AG	134.27	113.68	25.94	28.46
AT	65.09	54.52	53.13	53.39
CG	0.48	0.44	0.78	0.64
AAC	1.76	1.44	4.15	4.31
AAG	7.26	6.25	7.45	8.41
AAT	9.26	6.76	39.55	39.17
ACC	6.97	6.48	1.41	1.55
ACG	0.74	1.03	0.18	0.26
AAAC	0.33	0.27	0.22	0.25
AAAG	1.13	0.79	0.91	1.02
AAAT	4.58	2.76	4.09	4.2
AACC	0.1	0.05	0.03	0.02
AACG	0.01	0.01	0.03	0.04
AAAAC	0.14	0.11	0.04	0.07
AAAAG	0.46	0.34	0.32	0.22
AAAAT	0.57	0.35	0.46	0.47
AAACC	0.14	0.12	0.02	0.04
AAACG	0.02	0.01	0.02	0.03
AAAAAC	0.2	0.17	0.03	0.05
AAAAAG	0.22	0.08	0.08	0.1
AAAAAT	0.34	0.29	0.16	0.13
AAAACC	0.02	0.04	0	0
AAAACG	0	00.01	0
